# Le paludisme grave d’importation chez l’adulte: étude rétrospective de treize cas admis en réanimation à Marrakech

**DOI:** 10.11604/pamj.2016.25.179.8558

**Published:** 2016-11-21

**Authors:** El Mostafa El Mezouari, Ayoub Belhadj, Mohamed Ziani, Mohamed Boughanem, Redouane Moutaj

**Affiliations:** 1 Service de Parasitologie Mycologie, Hôpital Militaire Avicenne Marrakech, Maroc; 2 Service de Réanimation Médicale, Hôpital Militaire Avicenne Marrakech, Maroc; 3 Service des Maladies Infectieuses, Hôpital Militaire Avicenne Marrakech, Maroc

**Keywords:** Paludisme grave, Plasmodium falciparum, réanimation, Severe malaria, Plasmodium falciparum, resuscitation

## Abstract

Le paludisme d’importation est une affection de plus en plus fréquente en zone non endémique. Les formes graves représentent 10 % des cas de paludisme à *Plasmodium falciparum*. Au Maroc, plus de 50 cas de paludisme sont enregistrés chaque année dont 83 % à *P. falciparum*. Ont été inclus dans l’étude tous les patients ayant développé un paludisme grave, admis au service de réanimation durant la période comprise entre le 1er Novembre 2009 et le 31 décembre 2015. Les principales données épidémiologiques, les motifs d’admission, la prise en charge et l’évolution ont été étudiés. Treize patients sont retenus. L’âge moyen est de 31 ans. Tous les patients ont séjourné en afrique subsaharienne et étaient non-immuns. La chimioprophylaxie était adéquate dans 33% des cas. Le délai moyen entre le début des symptômes et l’instauration du traitement était de six jours. La parasitémie moyenne initiale était de 12 %. Les motifs d’admission en réanimation étaient un coma (15%), une convulsion (07%), une détresse respiratoire (07%), une prostration (07%), une insuffisance rénale (07%), un choc associé à un ictère et une acidose (07%) et enfin une insuffisance rénale conjuguée à un coma (07%). Tous les patients ont reçu un traitement par la quinine intraveineuse avec une dose de charge dans 100 % des cas. Le taux de mortalité était de 23 %. Les causes du décès étaient dues à la défaillance multi viscérale et au syndrome de détresse respiratoire aigu. La mortalité des formes graves du paludisme reste élevée. L’adéquation de la chimioprophylaxie associée à la précocité du diagnostic et du traitement permettrait d’améliorer significativement le pronostic de cette parasitose.

## Abstract (en)

Imported malaria is being seen with increasing frequency in non-endemic areas. Severe forms represent 10% of cases of Plasmodium falciparum malaria. In Morocco, more than 50 cases of malaria occur each year, 83% of which with Plasmodium falciparum malaria. All patients with severe malaria admitted to the Intensive Care Unit during the period between 1 November 2009 and 31 December 2015 were enrolled in our study. The main epidemiological data, the reasons for admission, the management and the outcomes of patients were studied. Thirteen patients were included in our study. The average age was 31 years. All patients had been living in sub-Saharan Africa and had no immunity to malaria. Chemoprophylaxis was adequate in 33% of cases. The mean time between symptom onset and treatment initiation was six days. Mean initial parasitemia was 12%. The main reasons for ICU admission included coma (15%), convulsion (07%), respiratory distress 07%), prostration (07%), renal failure (07%), shock associated with jaundice and acidosis (07%) and kidney failure associated with coma (07%). All patients were treated with intravenous quinine loading dose. Mortality rate was 23%. The causes of death were multi-system organ failure and acute respiratory distress syndrome. Mortality associated with severe malaria remains high. The adequacy of chemoprophylaxis associated with early diagnosis and treatment would significantly improve the prognosis of this parasitic infection.

## Introduction

Le paludisme est un enjeu majeur de santé publique par la fréquence et la létalité de ses formes graves liées à *Plasmodium falciparum*. Au Maroc, depuis la neutralisation du dernier foyer de *Plasmodium vivax* en 2004, sont enregistrés chaque année une centaine de cas de paludisme d’importation dont 83 % à *Plasmodium falciparum*, provenant dans la majorité des cas d’Afrique subsaharienne. Les populations concernées sont les expatriés et les migrants vivant au Maroc et retournant à leur pays d’origine et surtout les voyageurs marocains occasionnels en afrique subsaharienne [1]. La précocité du diagnostic et l’adéquation du traitement sont les facteurs essentiels du pronostic. La plupart des formes graves ou fatales surviennent en raison d’un retard de prise en charge, par négligence des patients ou de leur entourage et/ou du fait de confusions diagnostiques. Le but de notre travail est de décrire le profil épidémiologique, clinique et biologique des cas de paludisme grave colligés et d’analyser les critères de gravité, motifs d’admission des patients en réanimation.

## Méthodes

Nous rapportons une étude rétrospective descriptive portant sur 13 cas de paludisme grave, diagnostiqués au service de parasitologie mycologie et traités au service de réanimation de l’hôpital militaire Avicenne de Marrakech, durant la période comprise entre le 1er Novembre 2009 et le 31 décembre 2015. Les patients inclus dans notre étude avaient été hospitalisés au service de réanimation avec comme diagnostic principal ou secondaire un paludisme grave. Le diagnostic était confirmé par étude parasitologique positive du frottis sanguin mince et/ou de la goutte épaisse avec mise en évidence de *Plasmodium falciparum*. Les critères de gravité utilisés pour le diagnostic sont ceux définis par l’OMS en 2000 [2]: prostration; Trouble de la conscience : score de Glasgow modifié<10; Crises convulsives généralisées répétées (plus de 2 en 24 heures); Détresse respiratoire; Œdème pulmonaire: définition radiologique; Etat de choc (PAS< 8O mmHg et signes périphériques d’insuffisance circulatoire); Saignement anormal; Hémoglobinurie (urines rouge foncé, hémoglobinurie à la bandelette); Insuffisance rénale (créatinémie > 265µmol/l et/ou oligurie <400ml/j); Hypoglycémie (glycémie< 2,2 mmol/l); Anémie grave (Hb < 5g/dl ou Ht<15%); Acidose : bicarbonates < 15 mmol/l ± pH < 7,35; Hyperlactatémie: lactates plasmatiques > 5 mmol/l; Hyperparasitémie = 4% chez le non immun; Ictère (clinique ou bilirubinémie totale>50 µmol/l).

Les données épidémiologiques, biologiques, cliniques et thérapeutiques ont été recueillies à partir des registres et des dossiers médicaux des patients hospitalisés pour paludisme à *P. falciparum* comprenant l’âge, le sexe, la profession, le pays visité, la durée de séjour en zone d’endémie, le type de séjour (professionnel, tourisme, familial…), la chimioprophylaxie, le délai entre le retour et les premiers symptômes, le délai entre les premiers signes et la prise en charge (diagnostic et traitement), le délai d’hospitalisation en réanimation. La gravité à l’admission en réanimation notamment la sévérité initiale a été estimée par le calcul de scores de gravité en particulier Apache II et SAPS II. Les critères de gravité du paludisme de l’OMS ont été pris en compte à l’admission et au cours du suivi en réanimation. Les données parasitologiques et biologiques : frottis et goutte épaisse (espèces plasmodiales, stades parasitaires, parasitémie à l’entrée, évolution de la parasitémie sous traitement, bilan hématologique et biochimique. Les données thérapeutiques: le traitement antiparasitaire initial (durée et traitements adjuvants non spécifiques) et l’évolution (durée de séjour en réanimation, favorable et défavorable).

## Résultats

Tous nos patients sont de nationalité marocaine, nés et résident au Maroc. L’âge moyen est de 31+/-5 ans. Ils sont exclusivement de sexe masculin. Aucun antécédent pathologique n’a été reporté chez nos patients, hormis une notion d’allergie chez un patient. La durée moyenne de séjour en zone d’endémie était de 9,2 mois avec des extrêmes allant de 2 jours à 14 mois. Tous nos malades ont contracté la maladie sur le continent africain dans la zone subsaharienne. Sept patients avaient séjourné en république démocratique du Congo (RDC), quatre en côte d’Ivoire, un au Mali et le dernier en Guinée. 10 cas des sujets hospitalisés sont des militaires faisant parti du contingent marocain en RDC ou Cote d’ivoire, 1 cas est ouvrier de profession alors que les 2 autres cas sont des voyageurs ponctuels: 1 pour séjour touristique et l’autre pour un voyage d’affaire. La chimioprophylaxie était adaptée aux protocoles recommandés pour le pays visité chez 11 patients. Elle était à base de : méfloquine dans 7 cas (58%) et l’association chloroquine-proguanil (savarine^®^) dans 4 cas (34%). Cette chimioprophylaxie a été jugée adéquate chez 4 patients (33%). Le délai d’apparition des symptômes était renseigné seulement chez 9 malades (soit 69% des cas), il était de 21 jours ± 3 jours. Nos patients étaient admis au service de réanimation soit directement par le biais du service des urgences (5 cas soit 38%). Chez ces patients, le délai entre les premiers symptômes et l’admission en réanimation était de 7jours ± 3 jours. Soit après un séjour au service de médecine interne, d’une durée moyenne de 5 jours ±4 jours, le passage en réanimation correspondait à une aggravation secondaire (8 cas soit 62%) (Tableau 1). L’espèce plasmodiale principalement en cause était *Plasmodium falciparum* qui était la seule espèce impliquée dans (11 cas soit 84.60%) ou associée dans deux cas (soit 15.40%) au *Plasmodium ovale*. La parasitémie était élevée, elle était en moyenne de 12% ±5. Les scores de gravité non spécifiques en particulier Apache II et SAPS II étaient respectivement de 38 ± 7 et 35 ± 5 après 24 h d’hospitalisation. L’analyse des critères de gravité de l’OMS au cours des 4 premiers jours d’hospitalisation en réanimation montre que chaque patient avait au moins l’association de 4 critères de gravité établis pas l’OMS. Quatre patients (30%) avaient présenté au cours de l’évolution une détérioration neurologique avec un coma profond (score de Glasgow moyen à 5 ± 2). Trois patients (23%) ont présenté une détresse respiratoire, dont un a évolué vers un SDRA authentique (avec PaO2/FiO2 = 150 ± 20, des infiltrats radiologiques bilatéraux, sans signes d’insuffisance ventriculaire gauche). L’insuffisance rénale aigue (IRA) était observée chez 3 de nos patients (soit 23%) et a nécessité le recours à l’EER chez 2 patients. Une CIVD biologique était présente chez 1 seul patient (soit 7%) et s’est avérée symptomatique, marquée par des hémorragies digestives hautes à type de méléna et d’hématémèse, ayant nécessité plusieurs transfusions de culots globulaires. Un patient avait rapporté une notion de somnolence avec prostration et une asthénie intense. Par ailleurs, nous avons noté un cas d’hypoglycémie (8%), deux cas d’anémie sévère (Hb < 5 g/dl) (17%) et deux cas d’acidose métabolique (17%) avec un pH moyen de 7,15 ± 0,6. Une hyperparasitémie = 4% était observée chez tous les patients. De même, 5 de nos patients étaient ictériques (42%) (Ictère clinique et/ou biologique bilirubinémie > 50µmol/l) et 5 patients ont présenté une dégradation de l’état hémodynamique avec une défaillance circulatoire majeure (42%).

**Tableau 1:** Critères de gravité conduisant au transfert des patients depuis le service de médecine interne vers la réanimation médicale de l’hôpital militaire Avicenne - Marrakech

Critères de transfert en réanimation	Nombre de cas (pourcentage)
Détresse respiratoire	1(7%)
Coma	2(15%)
Prostration	1(7%)
Convulsion	1(7%)
Insuffisance rénale	1(7%)
Choc+ictère+acidose	1(7%)
Insuffisance rénale+coma	1(7%)

Aucun cas d’hémoglobinurie macroscopique n’était observé. Le délai de la mise en route du traitement antipaludique était estimé à 5 ± 2 jours. Le traitement était à base de quinine avec une dose de charge (DC) de 17mg/kg en quatre heures, puis relayé par une dose de 8mg/kg toutes les huit heures, jusqu’à négativation de la parasitémie qui était obtenue après 10 jours ± 2 en moyenne. La ventilation artificielle était entreprise chez 7 patients (52%): 6 comas profonds et 1 syndrome de détresse respiratoire aiguë. Le remplissage vasculaire et l’optimisation de la volémie étaient entrepris chez tous les malades, sous surveillance hémodynamique. Les amines vasoactives ont été indiquées chez 5 patients (42%). Dans 2 cas d’insuffisance rénale aigue, on a eu recours à l’épuration extrarénale (soit 15%). Les anticonvulsivants (benzodiazépines, valproate de sodium, …) ont été indiqués pour contrôler les convulsions chez 4 malades (30%) (Tableau 2). La transfusion de culots globulaires était indiquée chez 2 malades (15%) pour maintenir un taux d’hémoglobine suffisant. Chez certains patients, des manifestations graves n’ayant pas été observées au moment du diagnostic de paludisme grave, sont survenues au cours de l’évolution de ce même accès palustre. Certains étaient directement liés au paludisme et d’autres aux complications de la réanimation. Les complications faisant partie de la définition OMS 2000 du paludisme grave survenues au cours de l’accès sont Coma: 1 cas (7%), Etat de choc: 4 cas (30%) dont un dans le cadre d’une défaillance multi viscérale, syndrome de détresse respiratoire aigue: 1 cas (7%), convulsion: 1cas (7%), hypoglycémie: 1 cas (7%), acidose: 2 cas (15%) et insuffisance rénale aigue: 1 cas (7%). La principale complication liée à la réanimation était la pneumopathie nosocomiale essentiellement aux bactéries à Gram négatif chez 2 patients soit 15% des cas (un cas à *Acinetobacter baumanni* et un autre cas à *Pseudomonas aeruginosa*). Concernant la durée de séjour en réanimation, elle était en moyenne de 13 ± 5 jours.

**Tableau 2:** Traitements, évolution des patients au service de réanimation médicale

	Nombre (%)
**Traitement spécifique**	
Quinine intraveineuse	13(100%)
Dose de charge	13(100%)
Délai de négativité parasitémie (survivants)	5-10 jours
**Traitement non spécifique**	
Ventilation mécanique	7(53%)
Amines vasopressives	5(38%)
Epuration extra rénale	2(15%)
transfusion	2(15%)
Pneumopathies nosocomiales	2(15%)
Durée de séjour	13 ± 5 j
Mortalité	3(23%)

## Discussion

L’âge moyen de nos patients (31ans) est inférieur à celui rapporté dans l’étude de P. Corne, K. Klouche [3], mais reste comparable à celui observé dans l’étude rapportée par B. Charra et al. [4]. Une population jeune est plus dynamique, et donc plus exposée aux voyages, que ca soit pour les études, pour le travail ou encore pour le service militaire. La prédominance masculine notée dans notre étude est liée au type de recrutement au sein de la collectivité militaire, dominé essentiellement par une population masculine ainsi que par le comportement masculin qui est différent de celui chez la femme en termes de respect des mesures prophylactiques. Les données épidémiologiques au Maroc font état de 100% de contamination en Afrique Subsaharienne [5]. En effet, tous nos patients ont contracté la maladie sur le continent africain dans la zone intertropicale en RDC, en côte d’Ivoire au Mali et la Guinée. Comme dans la plupart des études rapportées sur le paludisme d’importation, les aspects épidémiologiques les plus souvent associés à la survenue d’une forme grave au cours du paludisme d’importation sont le statut non immun des patients [6], l’absence d’une chimioprophylaxie appropriée et le retard diagnostique [7]. La durée moyenne du séjour en zone d’endémie de nos patients était de 9,2 mois (02 jours à 14 mois). Le délai moyen entre le retour de la zone d’endémie et l’apparition des premiers symptômes dans notre étude était de 21 jours ± 2 jours. Le délai de prise en charge qui correspond au retard thérapeutique était en moyenne 5±2jours. Le retard diagnostique est la troisième caractéristique épidémiologique associée à un plus fort risque de développer un paludisme grave. Il a été rapporté que même dans les conditions les plus défavorables, aucun accès palustre du à P. falciparum n’évoluait vers un accès grave s’il était traité dés les premiers signes de l’accès palustre simple et à l’inverse, un accès palustre à P. falciparum non traité pouvait évoluer vers une forme grave de paludisme dans les 36 à 48 heures suivant l’apparition des symptômes [8]. Le diagnostic et le traitement précoce de l’accès palustre restent les seuls garants d’une évolution favorable [9].

Tous nos patients étaient d’origine marocaine, sans antécédent de séjour en zone d’endémie palustre. Ils sont donc non immuns et à haut risque de contracter une forme grave potentiellement mortelle [4]. Cette constatation renforce la thèse selon laquelle une protection immunitaire, même partielle, est acquise chez les sujets ayant vécu de nombreuse années au contact du parasite sans chimioprophylaxie [6]. S’agissant du protocole de chimioprophylaxie suivi dans notre étude, 33% de nos patients avaient déclaré suivre une chimioprophylaxie correcte. L’absence de chimioprophylaxie ou une chimioprophylaxie inadaptée sont des facteurs de risque pour l’évolution vers une forme grave de paludisme [2]. En effet, dans notre étude, 8% des patients n’avaient pas pris de chimioprophylaxie et 59% avaient une chimioprophylaxie inadéquate en raison d’un arrêt trop précoce et/ou d’une prise irrégulière. Le diagnostic biologique du paludisme est une urgence [10]. 100% des patients de notre étude ont bénéficié d’un frottis sanguin et d’une goutte épaisse. Toute fois, la nécessité d’une auto-évaluation annuelle des biologistes assurant le diagnostic du paludisme est à souligner [10]. La goutte épaisse et le frottis sanguin servent pour le diagnostic du paludisme et aussi pour le suivi du traitement et l’évaluation de la parasitémie [2]. Elles étaient réalisées aux jours 3,7 et 28 du début de traitement afin de détecter le taux de parasitémie. Un délai de négativité est signalé après 5 à 10 jours. Le taux de mortalité dans notre étude est bas (23%) comparé à l’étude de B. Charraa et al. [4]. Alors que ce taux est supérieur par rapport au taux rapporté par l’étude de P. Corne et al. Le diagnostic précoce et l’orientation rapide des malades en réanimation sont probablement les points fondamentaux dans la prise en charge du paludisme grave à *Plasmodium falciparum*.

## Conclusion

Le paludisme grave à *Plasmodium falciparum* est lié à une mortalité élevée. La plupart des décès étaient associés soit à un retard diagnostique soit à une chimioprophylaxie inadéquate soit aux deux. La prévention resterait le meilleur moyen pour éviter la survenue des formes graves. Elle devrait être basée essentiellement sur l’information des voyageurs et l’adéquation de la chimioprophylaxie associée à la précocité du diagnostic et du traitement.

### Etat des connaissances actuelles sur le sujet

- Le paludisme est une pathologie grave aussi bien dans les pays endémiques et dans les zones non endémique;
- Le grand défi est toujours la prise en charge des patients atteints de la malaria et particulièrement en cas du paludisme grave. Elle doit être pluri disciplinaire;
- La rapidité du diagnostic est fondamentale vu le taux de mortalité élevé chez les patients atteints du paludisme à Plasmodium falciparum.

### Contribution de notre étude à la connaissance

- L’expérience de l’hôpital militaire Avicenne de Marrakech en matière de prise en charge des patients atteints du paludisme grave;
- Cette expérience aidera les autres collègues médecins des pays loin des zones d’endémie palustre à améliorer les qualités de soins et de diagnostic;
- Un rappel des critères de gravité palustre et leur fréquence.
